# Genetic Insights Into Hypothalamic Hamartoma: Unraveling Somatic Variants

**DOI:** 10.1212/NXG.0000000000200180

**Published:** 2024-09-04

**Authors:** Lina Sami, Mathilde Chipaux, Sarah Ferrand-Sorbets, Marion Doladilhe, Christine Bulteau, Emmanuel Raffo, Sarah Rosenberg, Georg Dorfmuller, Rayann Checri, Jean-Madeleine De Sainte Agathe, Eric Leguern, Homa Adle-Biassette, Sara Baldassari, Stephanie Baulac

**Affiliations:** From the Institut du Cerveau-Paris Brain Institute-ICM (L.S., M.D., J.-M.D.S.A., E.L., Sara Baldassari, Stephanie Baulac), Sorbonne Université, Inserm, CNRS, Hôpital de la Pitié Salpêtrière; Department of Pediatric Neurosurgery (M.C., S.F.-S., C.B., E.R., S.R., G.D., R.C.), Rothschild Foundation Hospital EPICARE; Department of Medical Genetics (J.-M.D.S.A., E.L.), AP-HP, Sorbonne Université, Hôpital de la Pitié Salpêtrière; and Université de Paris Cité (H.A.-B.), service d’Anatomie Pathologique, AP-HP, Hôpital Lariboisière, DMU DREAM, Biobank BB-0033-00064, UMR 1141, INSERM, Paris, France.

## Abstract

**Objectives:**

Hypothalamic hamartomas (HHs) are rare developmental brain lesions associated with drug-resistant epilepsy and often subjected to epilepsy surgery. Brain somatic variants in genes affecting the Sonic hedgehog (Shh) and primary cilia signaling pathways have been implicated in approximately 50% of nonsyndromic HH cases. This study aims to characterize a new cohort of 9 HH cases and elucidate their genetic etiology.

**Methods:**

We recruited 9 HH cases including 8 nonsyndromic cases of which 4 were type IV HH. Genomic DNA was extracted from peripheral blood and surgical brain tissues, and somatic variants were investigated using high-depth whole-exome sequencing.

**Results:**

Pathogenic somatic variants in known HH genes (*GLI3*, *OFD1*, and *PRKACA)* were identified in 7 of the 9 cases. In addition, a 2-hit mutational event comprising a germline variant (predicted to impair kinase activity) and a somatic loss-of-heterozygosity was identified in *TNK2*, a gene encoding a brain-expressed tyrosine kinase.

**Discussion:**

Our findings reinforce the role of somatic variants in Shh and cilia genes in HH cases while also shedding light on *TNK2* as a potential novel disease-causing gene. This study emphasizes the increasing importance of brain mosaicism in epilepsy disorders and underscores the critical role of genetic diagnosis derived from resected brain tissue.

## Introduction

Hypothalamic hamartomas (HHs) are rare congenital (noncancerous) growths of variable size and location on the hypothalamus, occurring in approximately 1 in 200,000 children. Most cases are sporadic and nonsyndromic, but approximately 5% of HHs are associated with Pallister-Hall and oro-facio-digital type VI syndromes. Nonsyndromic HH often manifest with epilepsy, most commonly as drug-resistant gelastic seizures (laughing attacks with onset in infancy), with cognitive and psychiatric comorbidities.^1,2^ Neurosurgery can be pursued to resect/disconnect the hamartoma, resulting in favorable surgical outcomes in 78% of patients.^3^ Evidence supporting postzygotic mosaicism within HH tissue as a disease mechanism has been recognized since 2008 (reviewed in reference 4). Somatic variants in genes of the Sonic hedgehog (Shh) signaling pathway and its regulators (e.g., *GLI3*, a transcription factor, and *PRKACA,* a repressor of Shh) or encoding primary cilia proteins (e.g., *OFD1*) account for approximately 50% of HH cases.^4^

In this article, we assembled a cohort of 9 surgical HH cases and searched for somatic variants in paired brain-blood or brain-only DNA samples using whole-exome sequencing (WES). We confirmed the major role of somatic variants in Shh and primary cilia genes in HH etiology and identified *TNK2* as a novel putative disease-causing gene.

## Methods

### Patient Recruitment

We collected a cohort of 9 patients who underwent epilepsy surgery for HH-associated drug-resistant epilepsy at the Rothschild Foundation Hospital (Paris, France) between 2016 and 2022. Frozen and formalin-fixed tissues were obtained for research and neuropathology purposes. Genomic DNA was extracted from blood and frozen surgical tissue using standard procedures.

### Standard Protocol Approvals, Registrations, and Patient Consents

The study protocol was approved by the ethical committee of CPP Ile de France II (ID-RCB/EUDRACT-2015-A00671-48); written informed consent was obtained from all patients.

### Genetic Investigations

WES was performed by IntegraGen (France) targeting 500X (brain) and 100X (blood) for sequencing coverage. Somatic and germline variants were called using MuTect2 and GATK (v4.1), respectively (eMethods for bioinformatic pipelines). We searched for somatic variants present either exclusively in the brain or in both the brain and blood. For germline variants, we focused the analysis on 336 genes related to “hypothalamic hamartoma,” “epilepsy,” and “ciliopathy” (listed in eTables 1 and 2) and all genes located on chromosome 3q of the loss-of-heterozygosity (LOH) region (for patient ICM_212) (listed in eTable 3). Copy number variants (CNVs) and LOH events were analyzed with GATK.

### Structural Modeling of the TNK2 Protein

We used SWISS-MODEL^5^ to generate a 3D model of the active and inactive kinase domains of TNK2 wild-type or p.M171T mutant (UniProt Q07912: Ser94-Gln456), based on 2 crystal structure templates: the TNK2 kinase domain with the C-terminal SH3 domain for the inactive state (4HZS) and the TNK2 kinase domain for the active state (4HZR).^6^ An adenosine diphosphate was incorporated by superposing the 1ol5 structure. Visualizations were created using Mol*Viewer.^7^

### Data Availability

Data are available from the corresponding author on request.

## Results

### Clinical Features of the Cohort

**Figure 1:**
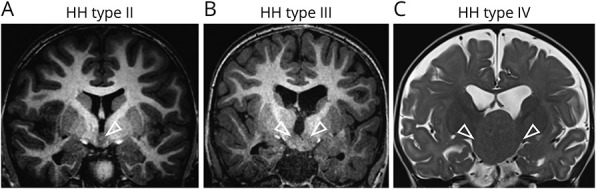
Representative MRI of HH Subtypes (A) MRI T1 coronal sequence of patient ICM_239 with HH type II attached to one side of the hypothalamus (white arrowhead). (B) MRI T1 coronal sequence of patient ICM_242 with HH type III attached bilaterally to the hypothalamus (white arrowheads). (C) MRI T2 coronal sequence of patient ICM_212 with a giant HH type IV with bilateral attachment to the hypothalamus (white arrowheads) and ventricular enlargement.

Patients' clinical features are summarized in the Table 1. The age at seizure onset ranged from birth to 2 years (median 3 months). Gelastic epilepsy (including gelastic epilepsy-plus with both gelastic and other seizure types), behavioral disorders, and cognitive/developmental impairment were observed in 9 of 9, 3 of 9, and 6 of 9 cases, respectively. HH type II (4 patients), III (1 patient), or giant IV (4 patients) were categorized according to the Delalande classification I-IV^8^ (Figure 1). 1 patient exhibited polydactyly and syndactyly. All HH type II cases had a good surgical outcome (Engel score I-II) while outcomes were less favorable in HH types III and IV (Engel score III-IV).

**Table 1:** Clinical Features and Genetic Findings of the HH Cohort

	ICM_242	ICM_246	ICM_264	ICM_239	ICM_55	ICM_194	ICM_186	ICM_212	ICM_171
Gene	*GLI3*	*GLI3*	*GLI3*	*GLI3*	*OFD1*	*OFD1*	*PRKACA*	*TNK2*	Unsolved
Variant (HGVSp)	p.I749Mfs*29	p.Q813*	p.L859*	p.E1147*	p.K240Gfs*8	p.G728Sfs*92	p.Y331_E332delins*	p.M171T	—
Variant (HGVSc)	c.2247_2250del	c.2437C>T	c.2575del	c.3439G>T	c.718_719del	c.2170_2177dup	c.993_995del	c.512T>C	—
Blood VAF	NA	0%	NA	0%	NA	0%	0%	49%	—
Brain VAF	26%	20%	25%	28%	58%	46%	19%	84%	—
HH type	III	II	IV	II	II	IV	IV	IV	II
Syndromic features	No	No	Polydactyly, syndactyly	No	No	No	No	No	No
Age at sz onset	0–6m	0–6m	0–6 m	1–2 y	6–12 m	Birth	Birth	Birth	1-2 y
Type of seizures	Gelastic epilepsy-plus (initial gelastic and dacrystic seizures at m3; complex focal seizures at y3)	Gelastic epilepsy-plus (initial gelastic and dacrystic seizures at m3; infantile spasms at m7)	Gelastic epilepsy-plus (initial gelastic seizures at m1; complex focal seizures at y11)	Gelastic epilepsy-plus (initial gelastic seizures at y2; complex focal seizures at y5)	Gelastic epilepsy	Gelastic epilepsy	Gelastic epilepsy (gelastic and dacrystic seizures)	Gelastic epilepsy (gelastic and dacrystic seizures)	Gelastic epilepsy only (from y1), one-year seizure free after first surgery at y2, then complex focal seizures (nongelastic)
No. of sz/day	2–3	2–3	2–4	6–9	5–8	4	80	70	2
Neuropsychological comorbidities	Developmental delay	Mild motor instability	Memory impairments	Hyperactivity, attention deficits	No	Mild behavioral disorder	Mild intellectual deficit, severe behavioral disorder, rage attacks	Mild intellectual deficit, behavioral disorder, rage attacks	Memory impairments, attention deficits, psychomotor delay
No. of surgeries	2	1	2	1	1	1	4	1	2
Age at surgery	7 y	1 y	11 y	5 y	10 y	5 y	7 y	1 y	22 y
Duration of the epilepsy	7 y	1 y	11 y	3 y	10 y	5 y	7 y	1 y	22 y
Engel score (FU)	III (6 y)	I (1 y)	I (1 y)	I (1 y)	I (6 y)	III (1 y)	I (8 y)	III (2 y)	I (2 y)

Abbreviations: del = deletion; dup = duplication; fs = frameshift; FU = follow-up duration; HGVSc = cDNA change annotation; HGVSp = protein change annotation; HH = hypothalamic hamartoma; ins = insertion; m = months; NA = not available; sz = seizures; VAF = variant allele frequency; y = years.

RefSeq references are *GLI3*: NM_000168.6, *OFD1*: NM_003611.3, *PRKACA*: NM_002730.4, and *TNK2*: NM_001382273.1. Disconnection was performed in all cases; patients ICM_212 and ICM_186 also had a surgical resection. The Engel score was used to classify the outcome of the epilepsy surgery. In case of multiple surgeries, age at surgery refers to the last surgery.

### Genetic Investigations

**Figure 2:**
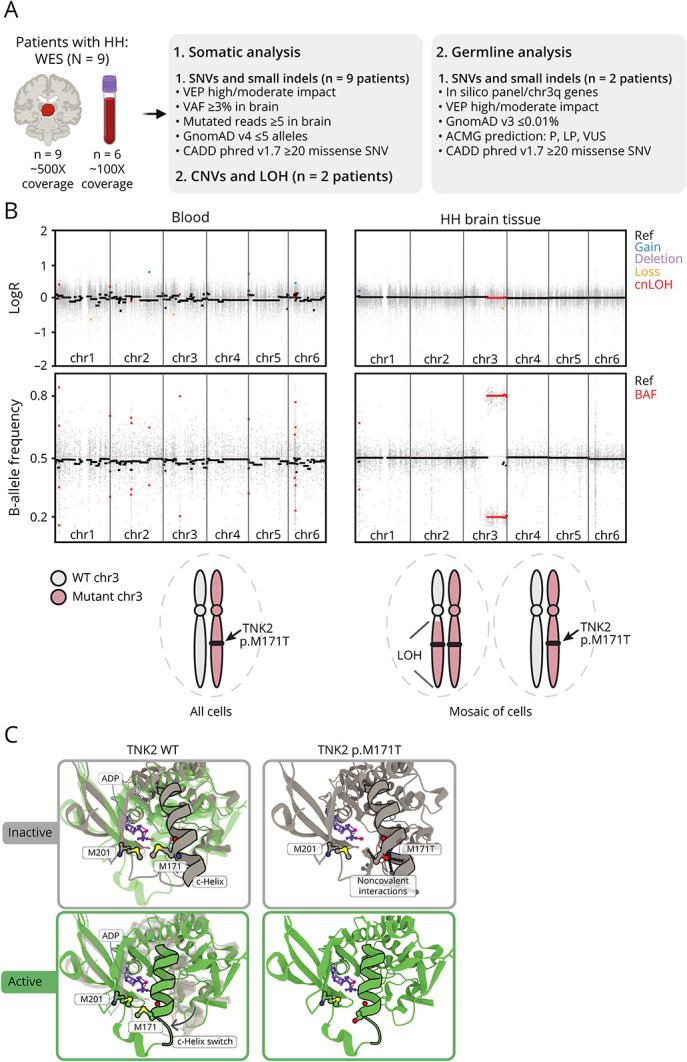
Two-Hit Genetic Event in *TNK2* and Protein Modeling of the p.M171T Variant (A) Whole-exome sequencing (WES) and bioinformatic analysis workflow. (B) Top: somatic loss-of-heterozygosity (LOH) on chromosome 3q (chr3q29) detected in HH brain tissue (but not in the blood) from patient ICM_212 (B-allele frequency distribution). The absence of chromosome gain or loss (LogR ratio) on chr3q indicates copy-neutral LOH (cnLOH). Bottom: schematic of wild-type (WT) and mutant TNK2 loci in blood and HH brain cells. (C) *In silico* 3D modeling of the inactive (grey) and active (green) states of the TNK2 kinase domain. The p.M171 residue lies within a C-helix, whose inward switch is required for the activation of kinase domains.^9^ In the WT configuration, the M171 points toward the M201, displaying hydrophobic interactions (pink halo) that are lost in the mutant TNK2. The p.M171T is predicted to destabilize the inactive state of TNK2. ACMG = American College of Medical Genetics and Genomics; ADP = adenosine diphosphate; BAF, B-allele frequency; CNVs = copy number variants; HH = hypothalamic hamartoma; indels = insertions/deletions; LOH = loss-of-heterozygosity; LP = likely pathogenic; P = pathogenic; Ref, reference; SNV = single-nucleotide variant; VAF = variant allele frequency; VEP = variant effect predictor (Ensembl); VUS = variant of unknown significance.

WES was conducted on paired brain-blood (6 cases) or brain-only (3 cases) DNA samples (Figure 2A). We identified somatic pathogenic truncating variants in previously reported HH genes in 7 of the 9 patients: 4 patients had variants in *GLI3*, 2 boys had X-linked variants in *OFD1*, and 1 patient carried a variant in *PRKACA*. Variant allele frequencies (VAFs) ranged from 19% to 58% (Table 1).

In 2 patients (ICM_212 and ICM_171), we did not identify any pathogenic or likely pathogenic somatic SNVs in the WES data. We then excluded pathogenic germline variants from a list of 336 genes related to “hypothalamic hamartoma,” “epilepsy,” and “ciliopathy” (eTables 1 and 2). Consequently, we investigated somatic CNV and LOH events. 1 case (ICM_171) remained unsolved. In patient ICM_212, we detected a brain somatic copy-neutral LOH (without loss/gain of genomic material) spanning the long arm of chromosome 3 (chr3q, Figure 2B**,** eTable 3). Two germline variants on chr3q displayed an enriched VAF in the brain tissue (VAF ∼80%) compared with the blood sample (VAF ∼50%): p.G908E in *FNDC3B* and p.M171T in *TNK2*. We considered *TNK2*, encoding a nonreceptor tyrosine kinase previously linked to infantile epilepsy,^10^ as a potential disease-causing candidate.

TNK2, also known as ACK1, is involved in various cellular processes, including cell proliferation, survival, migration, and adhesion^11^ and is highly expressed in the developing and adult human brain, including the hypothalamus. We generated an in silico 3D model of the inactive and active states of the TNK2 kinase domain, which showed that the p.M171T variant, located within the kinase domain,^9,12^ is predicted to alter noncovalent interactions, potentially altering the kinase activity (Figure 2C). Based on *TNK2* 2-hit genetic mechanism and insights from the 3D model, the p.M171T variant is presumed to act as a loss-of-function variant.

## Discussion

In this article, we report a cohort of 9 patients, 8 with nonsyndromic HH, including 4 with giant HH (type IV) and 1 with syndromic HH. Consistent with a recent large cohort study of 78 patients,^13^ we found gelastic seizures to be the most prevalent semiology (9/9). Our focused approach on brain somatic variants resulted in a diagnostic rate of 78% (7/9), beyond previous studies with diagnostic yields ranging from 32% to 51%.^4^

We identified somatic variants in the Shh and primary cilia pathways, with *GLI3* emerging as the most frequently mutated. In our cohort, all 4 patients with a *GLI3* variant had a gelastic epilepsy-plus phenotype. Type IV HHs were caused by variants in different causal genes, suggesting that the type of lesion may not be determined by the specific gene that is mutated. Further genetic studies in a larger cohort will enable genotype-phenotype correlations, helping determine whether certain HH genes are linked to specific clinical outcomes. In addition, we identified a 2-hit germline (p.M171T variant predicted pathogenic by in silico 3D modeling) and somatic (LOH) mutational event in *TNK2* gene in 1 patient. Biallelic *TNK2* germline variants have previously been reported in 2 unrelated patients with drug-resistant infantile spasms.^10^ The identification of additional *TNK2*-related HH cases and functional studies will be necessary to definitively establish *TNK2* as a novel HH-causing gene.

Somatic variants in HH predominantly target genes of the Shh pathway and primary cilia, potentially leading to decreased Shh signaling response and defective ciliogenesis; yet, the precise pathogenesis remains unclear. In the future, genetic testing using tissue samples from stereo-EEG electrodes may identify somatic variants during epilepsy presurgical evaluation, as previously reported in other epileptogenic malformations.^14-16^ This could pave the way for exploring personalized treatment strategies targeting the Shh signaling pathway, already in use in cancers,^17^ to HH cases with persisting seizures after initial surgery.

Although HH lesions are known to be intrinsically epileptogenic, the cell type and molecular mechanisms that drive HH lesion formation and epileptogenesis remain undefined. The generation of in vivo models reproducing somatic variants in the developing hypothalamus along with the use of single-cell approaches will allow for the assessment of these mechanisms. Our study highlights the importance of brain somatic mosaicism in epilepsy-associated neurodevelopmental disorders and underscores the importance of genetic diagnosis from resected brain tissue for precision medicine.

## Appendix Authors

**Table:**

Name	Location	Contribution
Lina Sami, MSc	Institut du Cerveau-Paris Brain Institute-ICM, Sorbonne Université, Inserm, CNRS, Hôpital de la Pitié Salpêtrière, Paris, France	Drafting/revision of the manuscript for content, including medical writing for content; major role in the acquisition of data; analysis or interpretation of data
Mathilde Chipaux, MD, PhD	Department of Pediatric Neurosurgery, Rothschild Foundation Hospital EPICARE, Paris, France	Drafting/revision of the manuscript for content, including medical writing for content
Sarah Ferrand-Sorbets, MD	Department of Pediatric Neurosurgery, Rothschild Foundation Hospital EPICARE, Paris, France	Drafting/revision of the manuscript for content, including medical writing for content
Marion Doladilhe, BSc	Institut du Cerveau-Paris Brain Institute-ICM, Sorbonne Université, Inserm, CNRS, Hôpital de la Pitié Salpêtrière, Paris, France	Analysis or interpretation of data
Christine Bulteau, MD, PhD	Department of Pediatric Neurosurgery, Rothschild Foundation Hospital EPICARE, Paris, France	Drafting/revision of the manuscript for content, including medical writing for content
Emmanuel Raffo, MD, PhD	Department of Pediatric Neurosurgery, Rothschild Foundation Hospital EPICARE, Paris, France	Drafting/revision of the manuscript for content, including medical writing for content
Sarah Rosenberg, MD, PhD	Department of Pediatric Neurosurgery, Rothschild Foundation Hospital EPICARE, Paris, France	Drafting/revision of the manuscript for content, including medical writing for content
Georg Dorfmuller, MD	Department of Pediatric Neurosurgery, Rothschild Foundation Hospital EPICARE, Paris, France	Drafting/revision of the manuscript for content, including medical writing for content
Rayann Checri, MD	Department of Pediatric Neurosurgery, Rothschild Foundation Hospital EPICARE, Paris, France	Drafting/revision of the manuscript for content, including medical writing for content
Jean-Madeleine De Sainte Agathe, MD	Institut du Cerveau-Paris Brain Institute-ICM, Sorbonne Université, Inserm, CNRS, Hôpital de la Pitié Salpêtrière; Department of Medical Genetics, AP-HP, Sorbonne Université, Hôpital de la Pitié Salpêtrière, Paris, France	Drafting/revision of the manuscript for content, including medical writing for content
Eric Leguern, MD, PhD	Institut du Cerveau-Paris Brain Institute-ICM, Sorbonne Université, Inserm, CNRS, Hôpital de la Pitié Salpêtrière; Department of Medical Genetics, AP-HP, Sorbonne Université, Hôpital de la Pitié Salpêtrière, Paris, France	Drafting/revision of the manuscript for content, including medical writing for content
Homa Adle-Biassette, MD, PhD	Université de Paris Cité, service d’Anatomie Pathologique, AP-HP, Hôpital Lariboisière, DMU DREAM, Biobank BB-0033-00064, UMR 1141, INSERM, Paris, France	Drafting/revision of the manuscript for content, including medical writing for content
Sara Baldassari, PhD	Institut du Cerveau-Paris Brain Institute-ICM, Sorbonne Université, Inserm, CNRS, Hôpital de la Pitié Salpêtrière, Paris, France	Drafting/revision of the manuscript for content, including medical writing for content; study concept or design; analysis or interpretation of data
Stephanie Baulac, PhD	Institut du Cerveau-Paris Brain Institute-ICM, Sorbonne Université, Inserm, CNRS, Hôpital de la Pitié Salpêtrière, Paris, France	Drafting/revision of the manuscript for content, including medical writing for content; study concept or design
